# *Non-Invasive* Acidic Pretreatment Technology of Anaerobic Digestion of Waste-Activated Sludge (WAS) on Biogas Production: Unveiling the Role of Extracellular Polymeric Substances (EPSs) and Pharmaceutical Degradation

**DOI:** 10.3390/molecules31020269

**Published:** 2026-01-13

**Authors:** Dragana S. Žmukić, Ljiljana Milovanović, Nataša Slijepčević, Nataša Duduković, Đurđa Kerkez, Lila Boudahmane, Emilie Caupos, Julien Le Roux, Régis Moilleron, Anita S. Leovac Maćerak

**Affiliations:** 1 Faculty of Sciences, University of Novi Sad, Trg Dositeja Obradovića 3, 21125 Novi Sad, Serbiaanita.leovac@dh.uns.ac.rs (A.S.L.M.); 2 LEESU, University Paris Est Creteil, ENPC, Institute Polytechnique de Paris, 94010 Creteil, Francejulien.le-roux@u-pec.fr (J.L.R.); moilleron@u-pec.fr (R.M.)

**Keywords:** anaerobic digestion, sludge, acidic treatment, EPS, pharmaceuticals

## Abstract

Non-invasive acidic pretreatments using acetic acid (1–5 mM) and citric acid (0.02–0.1 g g^−1^ TS) were investigated to enhance anaerobic digestion (AD) of waste-activated sludge (WAS). Both pretreatments improved short-term process stability, with pH (6.5–7.1) and alkalinity (1000–5000 mg CaCO_3_ L^−1^) remaining within optimal ranges during 10-day digestion. Acetic acid markedly enhanced solubilization and acidification, increasing volatile fatty acids to ~2500 mg L^−1^ (+67% vs. control), whereas citric acid achieved ~2000 mg L^−1^ (+37%). EPS analysis revealed pronounced redistribution of polysaccharides and proteins, with acetic acid inducing stronger disruption of the EPS matrix (SB-EPS polysaccharides up to 34.1 mg eq Glc mL^−1^). Specific methane yield increased from 28.5 mL CH_4_ g^−1^ VS (control) to 101.7 mL CH_4_ g^−1^ VS with acetic acid (3.6-fold) and to 73.8 mL CH_4_ g^−1^ VS with citric acid (2.5-fold). Gompertz modeling confirmed higher maximum methane potential, ~68% higher maximum methane production rates, and reduced lag phases for both pretreatments. In contrast, pharmaceutical concentrations (31 compounds) were largely unaffected by acid pretreatment, with significant reductions observed only for selected biodegradable molecules.

## 1. Introduction

Wastewater treatment processes result in the production of significant amounts of sludge, a complex mixture of microorganisms, extracellular polymeric substances (EPSs), and residual organic and inorganic matter. Among the organic substances present, pharmaceuticals are especially noteworthy due to how they enter the wastewater system and their tendency to accumulate and persist in the sludge [1,2]. Sludge management accounts for almost half of the total operational costs of wastewater treatment plants [3], yet it represents a potential resource when stabilized and valorized through anaerobic digestion (AD) [4].

Anaerobic digestion is a biological process that converts organic matter into biogas through four main stages: hydrolysis, acidogenesis, acetogenesis, and methanogenesis [5]. This is one of the most environmentally friendly methods in sludge management [6]. The problem with anaerobic digestion can be the decomposition of complex organic structures, which is affected by factors such as process conditions, sludge content, microbial communities, and many others. Hydrolysis is generally recognized as the rate-limiting step, as the complex structure of EPS limits the accessibility of organic substrates to microorganisms [7,8]. EPS, composed mainly of polysaccharides, proteins, lipids, and DNA, forms soluble (S-EPS), loosely bound (LB-EPS), and tightly bound (TB-EPS) layers, with TB-EPS being particularly resistant to degradation [9,10,11]. They produce various microorganisms which consume organic matter during the biological treatment of wastewater [12].

In order to overcome the existing shortcomings and to increase the yield of biogas, various pretreatments are applied. Pretreatments are commonly employed to enhance hydrolysis efficiency and biogas yield by disrupting EPS and increasing substrate accessibility [13]. There are various physical, chemical and biological pretreatments that are applied. Chemical pretreatments are based on the application of acids, bases and oxidizers [2,14]. Chemical pretreatments are among the most effective [15], but conventional acids such as H_2_SO_4_, HCl, and HNO_3_ are often corrosive and environmentally harmful [16]. In addition, there are various physical pretreatments, such as application ultrasonication, microwaves, electro-kinetic disintegration, that would improve the decomposition of organic matter [17]. Physical pretreatments have certain disadvantages such as high energy consumption and the inefficiency of the process. Biological pretreatments can include aerobic pretreatment, anaerobic pretreatment and the addition of enzymes that promote the disintegration of organic matter. The disadvantage of these pretreatments is that they can be very expensive and unprofitable [2]. One of the most critical parameters for the sustainable improvement of anaerobic digestion sludge is the achievement of energy and economic efficiency [18]. In addition, ecological performance methods are an important parameter [19]. Therefore, the pretreatments should meet several different parameters, which poses additional challenges when improving anaerobic digestion. In recent studies, various less invasive pretreatments have been emphasized. One of the more environmentally friendly ways of improving anaerobic digestion is the use of carbon-conductive materials such as biochar [20,21]. In addition, there are other ways to increase biogas yield using less harmful chemicals that could potentially enhance the anaerobic digestion of sludge. For this reason, attention has shifted toward environmentally benign, green or “non-invasive” acids.

Acidogenesis is one of the first phases of anaerobic digestion in which volatile fatty acids (VFAs) are produced. Monomers formed in the hydrolysis phase are converted into volatile fatty acids by acidogenic bacteria in the acidogenic phase. Fermentation products such as acetate can be used by methanogenic bacteria to produce methane [22]. The addition of acetic acid could potentially enhance VFA production, which would affect the methanogenic phase and methane production [23]. Also, depending on the concentration used, acetic acid is one of the less harmful chemicals [24], compared to other acids. Citric acid is a widely used carboxylic acid. It is most often used in the food and pharmaceutical industry. It is a cheap acid, and is harmless and easily degradable by microorganisms [25]. Citric acid effectively disrupts outer EPS layers, enhancing dewaterability and solubilization [26,27].

The objectives of the study are to (1) evaluate the effects of *non-invasive* acetic and citric acid pretreatments on WAS solubilization and hydrolysis; (2) investigate EPS disruption and redistribution of polysaccharides and proteins; (3) quantify improvements in methane production and AD kinetics during rapid 10-day BMP tests; (4) assess the impact on pharmaceutical micropollutant degradation; and (5) compare the effectiveness of acetic acid versus citric acid pretreatment. This work is, to the best of our knowledge, the first to explore the combined effects of such pretreatments on biogas yield, EPS degradation, and pharmaceutical occurrence in anaerobically digested waste-activated sludge.

## 2. Results and Discussion

### 2.1. Impact of Non-Invasive Acidic Pretreatments on Anaerobic Fermentation Performances

**Figure 1:**
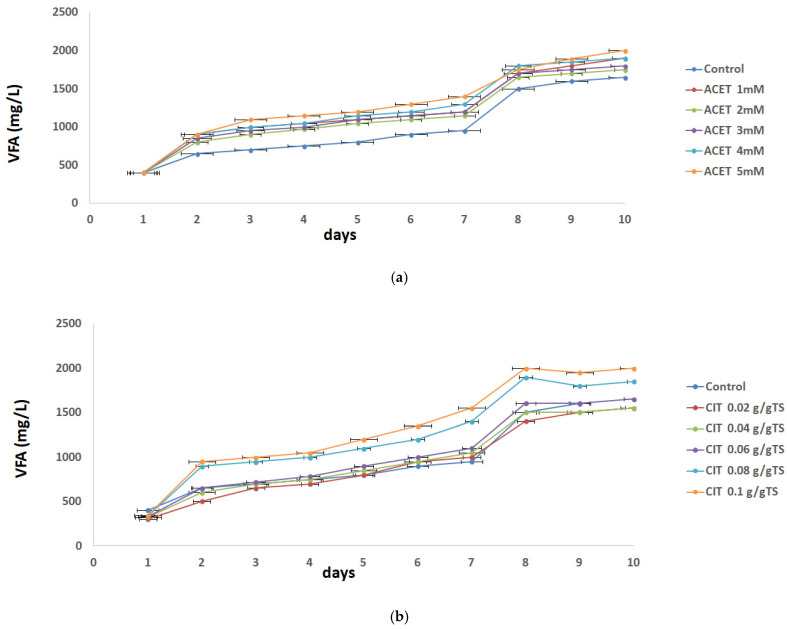
Effects of acid concentrations: (**a**) acetic acid 1 mM–5 mM and (**b**) citric acid 0.02–0.1 g/g TS on the VFA changes.

The stability parameters of the anaerobic digestion process are the environmental variables which affect the harmonic relationship between acid formers and methane formers during the AD process. Disturbance of these parameters affects the efficiency of methane formers, which causes the accumulation of acids in the reactor and may lead to process failure. Those factors are VFA concentration, pH, alkalinity and VFA/alkalinity ratio [28], and it should be noted that they reflect short-term process stability during 10-day digestion period. The AD performance from WAS pretreated with different concentrations of acetic and citric acid was first evaluated. Acidification is an important step in AD, and VFA, alkalinity and pH changes are the main parameters during the acidification process. As shown in Figure 1, VFA in the supernatant after AD increased with the increase in dosage of both acids, implying a positive effect of non-invasive acidic pretreatments on the anaerobic fermentation process. Concerning acetic acid pretreatment, VFA increased with the increase in digestion time following the same trend at all investigated dosages and reached a plateau after the eighth day. The VFA was around 2500 mg/L at the 5 mM acetic acid, which increased around 67% of that in the control test (around 1500 mg/L). Clearly, the addition of acetic acid improved the solubilization and hydrolysis efficacy of WAS. This performance was similar to that of the previous VFA production, which involved other pretreatments [29,30]. The VFAs displayed a similar tendency during citric acid pretreatment but exhibited lower VFA production in comparison to the acetic acid pretreatment. The VFA production was nearly 2000 mg/L at the highest citric acid concentration (0.1 g/g TS) and that enhancement was about 37% in comparison to the control test. One can draw the conclusion that overall acetic acid pretreatment was 1.8 times greater than with citric acid. The better performance of acetic acid in contrast to citric acid is expected due to chemical structure, biodegradability and microbial pathways. Citric acid is a larger, more complex tricarboxylic acid which is slowly degradable. In the presence of macronutrients (iron, calcium, magnesium) CA can chelate metal ions. Because of its mild antimicrobial characteristics, especially at low pH, CA can suppress hydrolytic or acidogenic bacteria needed for VFA production. But when it comes to practical application, CA may be more useful in controlled pH regulation [31].

**Figure 2:**
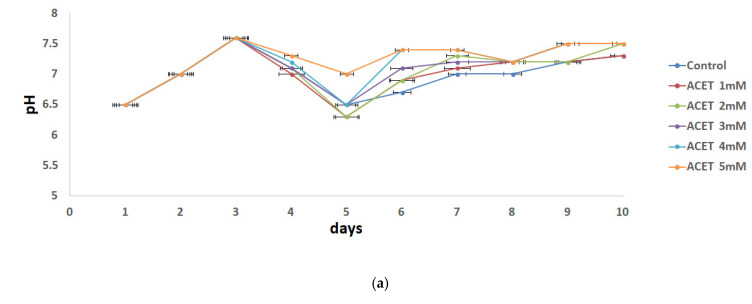

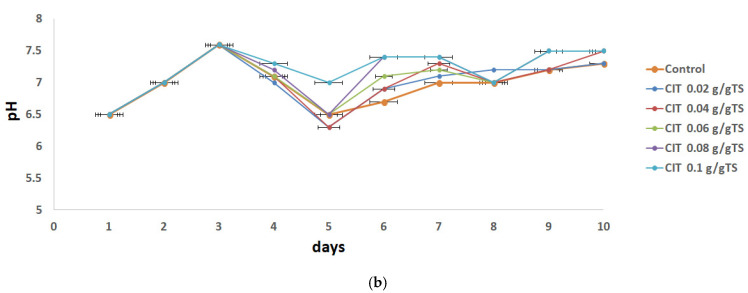
Effects of acid concentrations: (**a**) acetic acid 1 mM–5 mM and (**b**) citric acid 0.02–0.1 g/g TS on the pH changes.

The initial and final pH of all the bottles was measured as presented in Figure 2. Initial pHs for all the bottles were in the range of 6.5 to 7.6 which was in a suitable range for AD. The final pH of all bottles was in the range of 6.5 to 7.1. Methane-producing organisms require an optimal pH range of 6.5–8.2. When the pH value is higher than 8.5, it has toxic effects on the AD process.

**Figure 3:**
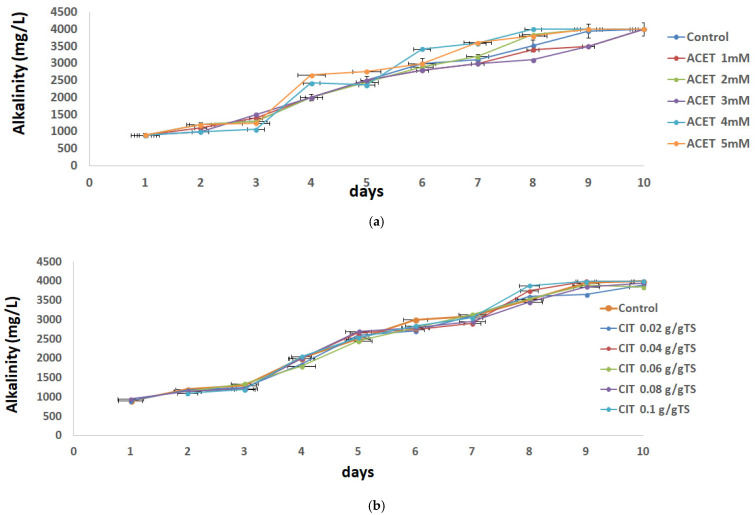
Effects of concentrations: (**a**) acetic acid 1 mM–5 mM and (**b**) citric acid 0.02–0.1 g/g TS on the alkalinity changes.

Apart from pH, alkalinity is another reliable parameter to assess the balance of the AD process. In this study, the only source of external alkalinity was inoculum. No additional chemical alkalinity was added in the bottles. The initial and final alkalinities of the reactor bottles are shown in Figure 3. The total alkalinity before the incubation ranged from 900 mg CaCO_3_/L to 4000 mg CaCO_3_/L, resulting in a good system performance, as the experiments remained within the optimal range of 1000 to 5000 mg CaCO_3_/L. This range of 1000 to 5000 mg CaCO_3_/L ensures the buffer capacity within the mixture and avoids large alkalinity variations. At the end of the experiment, CaCO_3_ concentration increased in all samples. As methane-forming bacteria consumed the volatile acids, alkalinity increased, and pH was kept stable, leading to positive effects on the AD conditions.

Additionally, 0.4 is thought to be a sign of healthy anaerobic digestion [32,33].

**Figure 4:**
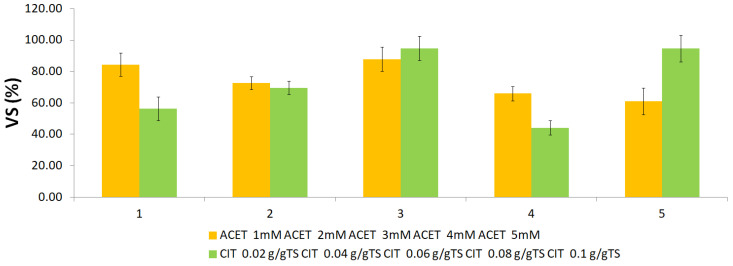
Effects of concentrations: acetic acid 1 mM–5 mM and citric acid 0.02–0.1 g/g TS S on the VS removal.

Angelidaki et al. [34] also consider that organic materials with VS content higher than 80% are susceptible to good degradability. When it comes to ACET treatment, the removal efficiencies were in the range 61.2–87.9%, while the removal range in the case of CIT acid was 44.2–94.7% (Figure 4).

### 2.2. Impact of Non-Invasive Acidic Pretreatments on Methane Production

**Figure 5:**
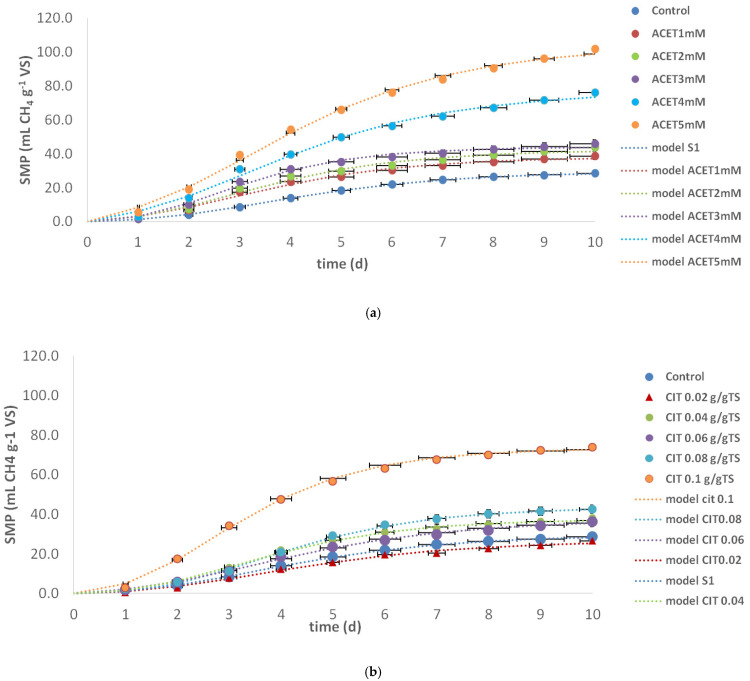
Cumulative specific methane production (SMP) with standard deviation for experimental data and the Gompertz model curve at different (**a**) acetic acid (1 mM–5 mM) and (**b**) citric acid (0.02–0.1 g/g TS) concentrations.

Figure 5a,b illustrates the impact of acetic acid and citric acid, respectively, on the methane production kinetics and the control condition without the addition of acids. The Gompertz model equation (Equation (1)) was fitted to the experimental specific methane production (SMP) obtained to estimate the kinetic parameters that describe the methane production, allowing a quantitative comparison between the studied conditions. Figure 5a,b shows cumulative daily SMP values, whereas the Gompertz model parameters are summarized in Table 1. The results were presented as means with the standard deviation of each determined parameter. The SMP production of WAS assessed under different concentrations of acetic acid (1–5 mM), which are presented in Figure 1a. Methane production was monitored for 10 days, reaching 28.5 mL CH_4_/g VS in control and from 38.6, 43.6, 45.8, 76.0 and 101.7 mL CH_4_/g VS for the acetic acid range of 1–5 mM, pointing to a significant increase with the increase with the addition of acetic acid in comparison to the control. Specifically, SMP increases from 35.4% to 256.8% with the increase in acetic acid concentration.

CIT treatment (Figure 5b) showed a similar increasing trend with the increase in CIT concentration: 26.6, 38.0, 36.3, 42,5 and 73.8 mL CH_4_/g VS for a range of 0.02–0.1 g/g TS. Based on Figure 5b, it could be indicated that significant variables were not observed at a lower CIT concentration. Specifically, SMP decreased by 7.2% at the dosage of 0.02 g CIT/g TS in comparison with the control. Additionally, a similar enhancement of SMP production of ~20% can be observed at concentrations of 0.04 and 0.06 g CIT/g TS. A significant increase of more than 30% is obtained at higher investigated concentrations > 0.08 g CIT/g TS.

The results showed that methane production of AD probes with ACET pretreatment at 1 mM–5 mM on digested sludge was effectively enhanced, with the highest production being 3.56 times of that observed in the control AD without ACET addition. When it comes to CIT pretreatment of AD probes, improved methane production was achieved at higher CIT concentration 0.08 and 0.1 g CIT/g TS, with the highest SMP of 2.59 in comparison to the control sample. Tang et al. [27], Amnuaycheewa et al. [35], and Garcia Gomes et al. [36] came to similar conclusions when investigating the same organic acids’ pretreatments’ effect on the AD of sewage sludge, sugarcane bagasse and rice straw, respectively.

The maximum specific methane production (M_∞_) obtained from the Gompertz model shows great fitting between experimental and modeled results. One-way ANOVA revealed a significant effect of acid pretreatment on the maximum methane potential (M_∞_) (*p* < 0.05), following the order acetic acid > citric acid > control, with all pairwise differences being statistically significant. M_∞_ obtained in the presence of the highest acetic acid concentration of 5 mM (104.1 mL CH_4_ g^−1^ VS) is 3.58 times higher than that in the control (29.1 mL CH_4_ g^−1^ VS). Thus, the addition of acetic acid had an impact on methane production during the anaerobic digestion of WAS. Acetic acid pretreatment could mechanistically influence AD and biogas yield because of organic matter solubilization acting as a weak acid by lowering the pH of the sludge matrix. In this way, ACET pretreatment disrupts EPS and microbial flocs, easily releases biodegradable organics and makes more available proteins, carbohydrates and lipids (through hydrolysis step) for fermentative and methanogenic bacteria. Acetic acid is not only a pretreatment reagent but also a direct substrate for methanogens, particularly acetoclastic methanogens, which convert acetate into methane (CH_4_) and CO_2_. In this way, the upstream fermentation of more complex compounds is omitted, providing a direct source of methanogenic substrate. Acetic acid has a mild buffering effect at low concentrations, helping stabilize pH during early fermentation, maintaining conditions favorable for acidogens and acetogens. Introducing acetic acid reduces the need for upstream fermentation processes and promotes acetoclastic methane production.

In the case of citric acid pretreatment, M_∞_ obtained in the presence of the highest citric acid concentration of 0.1 g/g TS (73.3 mL CH_4_ g^−1^ VS) is 2.52 times higher than that in the control (29.1 mL CH_4_ g^−1^ VS). Citric acid also disrupts EPS structure by chelating divalent cations (like Ca^2+^, Mg^2+^) that stabilize EPS matrices and lowering pH, which solubilizes proteins and polysaccharides, making substrates more bioavailable for acidogenic and methanogenic microbes. Gayathri et al. [37] used citric acid to remove EPS from waste-activated sludge, followed by ultrasonic pretreatment. Methane yield increased significantly (up to 0.43 L/g VS). Citric acid is a weak triprotic acid and contributes to the acidification of the sludge, lowering pH. In moderate doses, this shifts fermentation pathways, enhancing VFA production, particularly acetic acid, the main precursor of methane via acetoclastic methanogenesis. Citric acid can also serve as a carbon source, which is fermented into VFAs by acidogenic bacteria.

The maximum methane production rate (μ_max_) was another parameter used to describe the methane production kinetics. One-way ANOVA showed that acid pretreatment had a significant effect on the maximum methane production rate (µmax) (*p* < 0.05), with both acetic and citric acid pretreatments significantly increasing µmax compared to the control, while no significant difference was observed between the two acids. A positive impact of the addition of both acids was detected. The μ_max_ was increased by 67.8% and 68.2% by acetic and citric acid, respectively, compared with the maximum production rate obtained in the control, with values of 16.4 and 16.2 mL CH_4_ g^−1^ VS d^−1^, respectively. On the other hand, the lag phase decreased in both pretreatments, indicating the acceleration of methanogenesis. The lag phase (λ) was significantly affected by acid pretreatment (*p* < 0.05), with acetic acid pretreatment resulting in a significantly shorter lag phase compared to the control, indicating the accelerated onset of methanogenesis. The increased values of M_∞_ and μ_max_ on one side, and the decreased value of lag phase on the other side, indicate the prevalence of methanogenesis in relation to other processes.

### 2.3. Degradation of EPS During Non-Invasive Acidic Treatments

**Figure 6:**
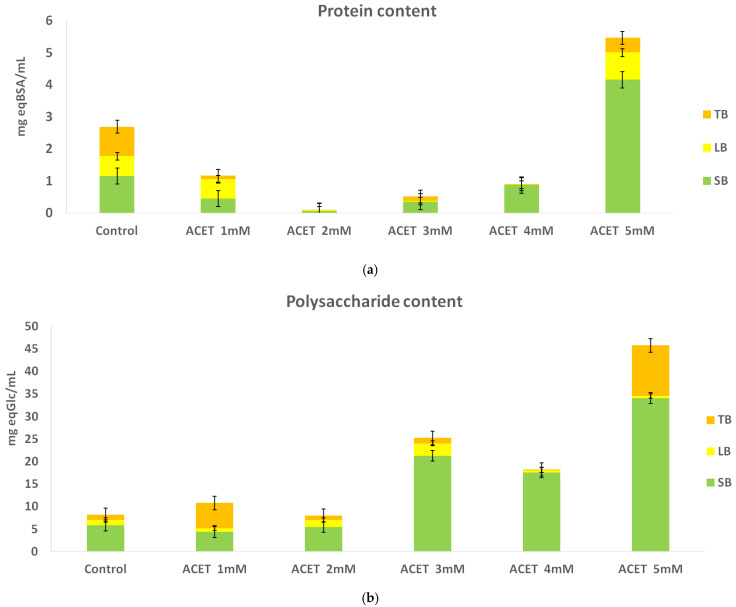
The content of total (**a**) protein and (**b**) polysaccharide in EPS fractions during acetic acid (1–5 mM) pretreatment.

Figure 6 summarizes the total polysaccharide and protein concentrations in the extracellular polymeric substance (EPS) fractions following pretreatment with acetic acid. In the SB-EPS fraction, polysaccharide content increased (*p* < 0.05) in all treatments except the one with the lowest acid concentration, where the measured concentration was 4.318 ± 0.108 mg eq Glc/mL, compared to 5.770 ± 0.661 mg eq Glc/mL in the untreated control. As the concentration of acetic acid increased, polysaccharide concentration in SB-EPS rose steadily, reaching a maximum of 34.10 ± 0.344 mg eq Glc/mL. Throughout all acetic acid treatments, SB-EPS consistently contained higher polysaccharide levels than LB-EPS, which likely reflects the transfer from LB-EPS to SB-EPS as part of a microorganism-mediated stress response to acid addition [38]. Within the LB-EPS fraction, polysaccharide concentrations generally exceeded control values except at the highest acid dosage, where a decline was observed. LB-EPS polysaccharide content was significantly affected by acid pretreatment (*p* < 0.05). The TB-EPS layer exhibited a modest increase relative to the control; however, at maximum acetic acid concentration, the increase became statistically significant (11.20 ± 1.210 mg eq Glc/mL). These trends may be attributed to the comparatively greater resistance of TB-EPS to breakdown relative to the outer layers. Acetic acid pretreatment significantly influenced SB-EPS protein content (*p* < 0.05), especially at higher dosages. Protein content decreased across most acid treatments in all EPS fractions, except at minimal and maximal acid volumes. When minimal acid was applied, protein concentration increased in the LB-EPS fraction (0.600 ± 0.000 mg eq BSA/mL), which also exhibited higher protein levels than the SB-EPS and TB-EPS layers. At the maximal acid dose, protein content increased in both SB-EPS (4.149 ± 0.340 mg eq BSA/mL) and LB-EPS (0.851 ± 0.012 mg eq BSA/mL). LB-EPS protein content differed significantly among treatments (*p* < 0.05), with acetic acid causing a substantial redistribution of proteins from tightly bound to loosely bound EPS fractions.

These results suggest that acetic acid-induced environmental stress may drive biomolecular redistribution among EPS layers. Although the relationship between acid concentration and biomolecular content is not uniformly linear across all fractions, the findings indicate that acetic acid pretreatment can aid the disruption of the rigid EPS matrix, potentially enhancing biodegradability and structural breakdown.

**Figure 7:**
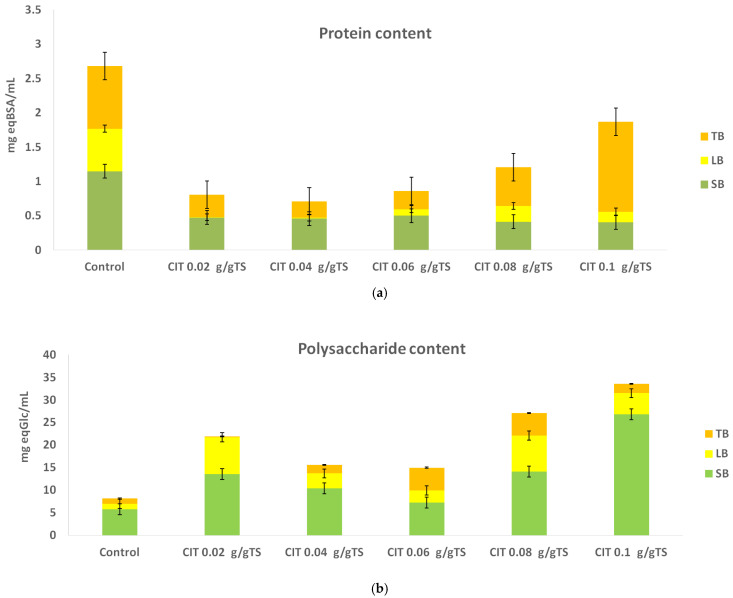
The content of total (**a**) protein and (**b**) polysaccharide in EPS fractions during citric acid (0.02–0.1 g/g TS) pretreatment.

Citric acid also significantly enhanced SB-, LB-, TB-polysaccharide release compared to the control (*p* < 0.05). Following citric acid pretreatment (Figure 7), polysaccharide concentrations in the SB-EPS fraction increased across all samples; similar to the response observed under acetic acid, SB-EPS consistently contained the highest polysaccharide levels among EPS layers. At the lowest citric acid concentration (0.02 g/g TS), the LB-EPS fraction exhibited a notable increase, reaching 8.125 ± 0.214 mg eq Glc/mL. As citric acid concentration increased, LB-EPS polysaccharide content fluctuated, ultimately decreasing to 4.697 ± 0.167 mg eq Glc/mL at the highest acid dose. The TB-EPS fraction mirrored this pattern: initial increases in polysaccharide content were followed by a decline at the maximum citric acid mass. These findings align with previously reported effects of citric acid pretreatment at 0.05 g/g suspended solids and an initial pH of 4, where LB-EPS content increased (from ~2.27 to ~5.49 mg/g VSS), while TB-EPS content decreased significantly (from ~12.35 to ~5.01 mg/g VSS), indicating effective disruption of outer EPS layers by citric acid [26]. Citric acid pretreatment induced significant changes in protein content across EPS fractions (*p* < 0.05), suggesting the partial disruption and redistribution of proteins within the EPS structure. In terms of protein content, increasing citric acid concentration corresponded with elevated protein levels in LB-EPS, whereas protein levels in SB-EPS and TB-EPS generally declined, except at the maximum acid dose, where TB-EPS protein concentration surpassed that of the control. Prior studies indicate that citric acid can induce sharp increases in both protein and carbohydrate across EPS fractions, accompanied by substantial damage to the TB-EPS layer, which may convert into LB-EPS and soluble organic matter [39], a mechanism consistent with the observed increase in LB-EPS biomolecules in the present experiment.

Overall, these results suggest that citric acid pretreatment selectively disrupts the outer EPS structure, promoting redistribution of polysaccharides and proteins toward the LB-EPS layer under conditions of acid-induced stress.

One can conclude that citric acid generally shows a weaker effect on protein decomposition than acetic acid in anaerobic sludge pretreatments due to different capacity to release undissociated protons; because acetic acid is a monoprotic weak acid (pK_a_ ≈ 4.8), it maintains a relatively high fraction of its undissociated form (CH_3_COOH) at pretreatment pH ≈ 2–4, which allows for effective intracellular diffusion and acid-catalyzed cleavage of structural proteins and cell walls, without being significantly sequestered by buffering or metal complexation. By contrast, citric acid is triprotic, with dissociation constants pK_a1_ ≈ 3.13, pK_a2_ ≈ 4.76, pK_a3_ ≈ 6.40. Within the same pH range, most citrate exists in dissociated ionic forms (H_2_Cit^−^, HCit^2−^, Cit^3−^), resulting in a much smaller proportion of undissociated acid molecules capable of transferring protons across microbial envelopes. Moreover, citrate has marked buffering capacity (effective over pH ≈ 2–7) and strong metal-chelation properties, both of which reduce the availability of free protons needed for hydrolysis, thereby inhibiting protein breakdown relative to acetic acid pretreatment [11,40,41,42]. Secondly, at lower dosages (e.g., 0.02–0.05 g citric acid per g TS), citric acid can deconstruct humic–protein complexes, releasing some hydrolases into solution—but this process is followed by a rebinding of released humics to protein/hydrolases, which can inhibit further hydrolysis over time. Acetic acid, being a smaller volatile VFA, does not foster such readsorptive behavior and thus may support more sustained protein solubilization [27]. Thirdly, citric acid serves as a readily degradable organic acid and metal chelator, which may act as a preferential microbial substrate. Analogous to how the presence of lactose typically suppresses protease/deamination activity in protein-rich sludge (by shifting microbial metabolism toward carbohydrate fermentation), citrate may similarly dampen protease activity and delay protein breakdown despite the same net pH effect. Acetic acid, on the other hand, is both a protein hydrolysis product and a direct methanogenic substrate, aligning with microbial pathways that prolong deamination and protein turnover [43]. Finally, citric acid is effective at deflocculating EPS and flushing them out of flocs, but it may not specifically solubilize protein fractions within SB-EPS, LB-EPS or TB-EPS fractions unless applied at higher doses or elevated temperatures. Acetic acid, due to stronger proton activity and VFA potency, often yields broader disruption, including protein liquor release [41].

### 2.4. Impact of Non-Invasive Treatments on the Removal of Pharmaceuticals

**Figure 8:**
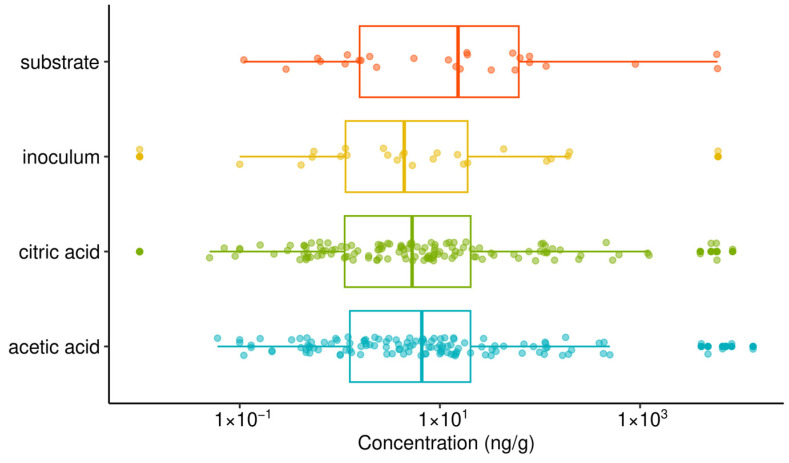
Concentrations of all pharmaceuticals analyzed in each type of sample of acidic pretreatment (boxplots and individual values).

A total of 31 pharmaceuticals were analyzed in anaerobically digested wastewater sludge. Most of the molecules were present at low concentrations, i.e., below 10 ng/g on average (e.g., acetaminophen, atenolol, amoxicillin, amisulpride, ketoprofen, irbesartan, naproxen). Some molecules were not detected (e.g., ranitidine, trimethoprim, sulfamethoxazole, clothianidine). Other specific molecules were present at concentrations between 20 and 70 ng/g (diclofenac) or around 100 ng/g (tramadol, penicillin), and some exhibited various concentrations depending on the samples (e.g., iopromide showed concentrations between 30 and 900 ng/g, with an average around 200 ng/g, and ciprofloxacin reached 13,000 ng/g in one specific sample while ranging between 50 and ~1000 ng/g in other samples). Ibuprofen was the most concentrated pharmaceutical in most samples, reaching concentrations of 4 to 11 µg/g. Similarly high concentrations of ciprofloxacin (and of other fluoroquinolones) have also been reported in various kinds of sludges [44]. Large concentrations of ibuprofen have also been reported, though not reaching the range observed in our study (e.g., 100–600 ng/g) [45]. These concentrations in sludge can be related to several factors: first, their occurrence in raw wastewater (either in the dissolved or particulate phase, depending on their consumption rate by the population and their excretion rate in urine and feces), the hydrophobicity of the molecules (governing their partition in the particulate phase through sorption into sludge), as well as their removal potential by anaerobic digestion (or in activated sludge). In particular, the high concentrations of ibuprofen, tramadol or penicillin can be related to their high hydrophobicity. Conversely, acetaminophen or sulfamethoxazole have lower logP values (indicative of their hydrophobicity), which can explain their lower presence in sludge despite usually high consumption. In addition, the large biodegradability of some molecules, including acetaminophen, can also explain their lower concentrations in sludges because of their degradation, either in biological treatment or in sludge treatment. No significant difference was observed between the range of concentrations of pharmaceuticals analyzed after each type of acid pretreatment (Figure 8). The concentrations of each individual molecule were not significantly different between the two pretreatments as well, except for amisulpride and citalopram, which showed lower concentrations after acetic acid pretreatment (*t*-test with *p*-value < 0.05). Overall, the range of concentrations was also similar between the substrate sample (before AD) and the acid-treated samples, showing a limited impact of the process on the degradation of organic micropollutants despite the enhanced anaerobic digestion. However, for 12 individual molecules (acetaminophen, amisulpride, atenolol, caffeine, carbamazepine, ciprofloxacin, citalopram, diuron, furosemide, iopromide, ketoprofen, naproxen) among the 26 detected organic compounds, the concentrations after acid pretreatment were much lower than the concentration before AD, indicating a substantial degradation of these molecules during the process, which can also be explained by their higher biodegradability.

## 3. Materials and Methods

### 3.1. Chemicals and Sludge Source

The acetic acid and citric acid were purchased from Sigma Aldrich (>95% purity). All pharmaceutical standards were of analytical grade, and stock solutions as well as calibration solutions were prepared in LC/MS-grade methanol (Thermo Fisher Scientific, Illkirch Cedex, France) [46]. The waste-activated sludge (WAS) used as a substrate and the anaerobic sludge supernatant used as inoculums were acquired from the WWTP (120,000 population equivalent) in Republic of Serbia. Table 2 summarizes the main characteristics of WAS and the inoculums.

### 3.2. Biochemical Methane Potential Tests

Glass serum bottles with a volume of 300 mL (150 mL working volume) were used to carry out the biochemical methane production (BMP) tests, with inoculums/substrate (I/S) of 2 (in VS) as detailed [47]. Nitrogen gas was used to purge each bottle before being sealed. BMP bottles were placed in a 37 °C incubator and shaken at 100 rpm to ensure mixing. Biogas production was measured daily using the classical volumetric displacement method. The BMP assays were conducted over a 10-day digestion period, which was intentionally selected to assess the early-stage effects of acidic pretreatments on anaerobic digestion kinetics, including methane production rate and lag phase, rather than to determine ultimate methane potential. Shortened BMP durations have been widely applied in pretreatment studies to evaluate process acceleration and enhanced substrate availability during the initial digestion phase [48,49,50]. Specific methane production (SMP) (mL CH_4_/g VS) was reported at normal conditions (*p* = 1 atm and T = 273 K). Inoculum blank bottles were prepared to determine the endogenous methane production. All tests were conducted in triplicate, and the results are expressed as their means with their standard deviations (SDs). The citric acid (CIT) concentration was in the range 0.02–0.1 g/g TS, while the acetic acid (ACET) concentration was in the range of 1–5 mM. The dosages of acids were selected to enable a comparative laboratory-scale assessment of non-invasive acidic pretreatments under controlled anaerobic digestion conditions. Dosage ranges for weak organic acids were chosen to achieve measurable effects on sludge solubilization and fermentation while minimizing potential inhibitory effects on methanogenesis. Moderate citric acid doses in the range of 0.02–0.10 g g^−1^ TS have been shown to enhance sludge solubilization and hydrolysis in anaerobic digestion, with higher dosages improving these effects without significant inhibition [27]. More broadly, acid pretreatments are reported to improve hydrolysis and organic release when appropriately dosed, balancing digestibility benefits and acid loading [51].

### 3.3. Methane Production Modeling

To describe and compare quantitatively the experimental data on methane production, we used the Gompertz mathematical model (Equation (1)), a modified sigmoid curve previously reported to describe the methane production of the degradation of simple substrates with an initial lag phase [47]. The Excel solver function estimated three different parameters by fitting the experimental data and the theoretical model using minimum squared error methodology. All experiments were performed in triplicate. Statistical differences among treatments were assessed using one-way analysis of variance (ANOVA, Minitab Statistical Software, version 21) followed by Tukey’s post hoc test, with statistical significance set at *p* < 0.05. Each parameter was represented in the model as follows: (i) maximum specific methane production (mL CH_4_ g^−1^ VS) as M_∞_; (ii) maximum methane production rate (mL CH_4_ g^−1^ VS d^−1^) as μmax; (iii) lag phase as λ (days).

(1) $$M\left(t\right)=M_{\infty }\cdot e\{-e\cdot \left[\frac{\mu_{max}\cdot e}{M_{\infty }}\cdot \left(\lambda -t\right)+1\right]\}$$

### 3.4. EPS Extraction

A heat extraction method [52,53] was applied to extract extracellular polymeric substances (EPS) from the sludge. The sludge suspension (30 mL) was centrifuged (5000× *g*, 15 min, 4 °C), and the supernatant was separated. The obtained supernatant contained soluble EPS (SB-EPS). The sludge pellet was re-suspended to 30 mL, in 0.05% (*w*/*v*) NaCl solution preheated at 70 °C. The suspension was vortexed for 1 min, and then centrifuged (5000× *g*, 10 min, 4 °C). Supernatant was designated as LB-EPS (loosely bound EPS). The left sludge pellet was again re-suspended to 30 mL in preheated (70 °C) 0.05% NaCl solution, and vortexed for 1 min. After incubation in a water bath (60 °C, 30 min), the suspension was centrifuged (5000× *g*, 10 min, 4 °C). The collected supernatant was regarded as tightly bound EPS (TB-EPS). The SB-EPS, LB-EPS and TB-EPS were filtered using 0.45 μM filters before analyzing for proteins and polysaccharides.

### 3.5. Analytical Methods

Total solids (TS), volatile solids (VS), chemical oxygen demand (COD) were quantified as described in APHA (2017) [54]. Carbon and nitrogen content (%) were obtained using Vario MACRO cube analyzer (Elementar, Germany; software version V3.1.1 (f224d35)). Alkalinity (expressed as mg CaCO_3_/L) and VFAs were determined by the titration method using 0.1 N NaOH and 0.1 N HCl [55]. The polysaccharide concentration was measured using the phenol–sulfuric acid method [56], with glucose as the standard. Protein concentration was determined according to the Lowry method [57], adapted to 96-well plates, and using bovine serum albumin (BSA) as the standard. The results were expressed as milligram equivalents of glucose per milliliter of extract (eqGlc/mL) ± SD for total polysaccharide content, and milligrams of BSA equivalents per milliliter of extract (eqBSA/mL) ± SD for total protein content. Additionally, it is important to highlight that all selected treatments were conducted in triplicate to ensure statistical reliability. These low standard deviation values indicate the good reproducibility of the experimental procedure and stability of treatment conditions, as well as reliable quantification of the investigated parameters. The analysis of 31 pharmaceuticals was conducted using ultrahigh-performance liquid chromatography (UPLC) coupled with tandem mass spectrometry (MS/MS), employing a triple quadrupole detector (Acquity-TQD, Waters, Milford, MA, USA) equipped with an electrospray ionization (ESI) source. Sludge samples were subjected to a modified QuEChERS extraction method inspired by Cuñat et al. [58], followed by an automated solid-phase extraction (SPE) step (Dionex Autotrace 280, Thermo Scientific, Waltham, MA, USA). Briefly, a 1 g sample of freeze-dried sludge was extracted with a mixture of methanol (5 mL), Milli-Q water (10 mL) and McIlvaine-EDTA solution (5 mL), after the addition of 4 g MgSO_4_, 1 g NaCl and internal standards. After agitation (1 h), the supernatant was recovered, and 10 mL of methanol was added to the remaining sample for additional extraction (with a 1 h agitation time). The final supernatants were combined and filtered (GF/F filter), then diluted in 500 mL of Milli-Q water. The solution was then extracted by SPE using homemade multilayer cartridges, as previously described for aqueous samples [59,60]. Specific analytical parameters, including stationary and mobile phases, internal standards, elution gradient, ionization mode, and MS/MS acquisition parameters, were previously described [46].

## 4. Conclusions

The results demonstrate that green, *non-invasive* acidic pretreatments effectively enhanced the anaerobic digestion performance of WAS by promoting solubilization and maintaining stable operational parameters.

Non-invasive acidic pretreatments significantly enhanced methane production kinetics during the rapid 10-day anaerobic digestion of WAS, with acetic acid showing a markedly stronger effect than citric acid. The highest SMP reached 101.7 mL CH_4_ g^−1^ VS with 5 mM acetic acid (≈3.6-fold higher than the control), while citric acid achieved a maximum of 73.8 mL CH_4_ g^−1^ VS at 0.1 g g^−1^ TS (≈2.5-fold increase). Gompertz modeling confirmed significantly higher maximum methane potential (M_∞_), increased maximum methane production rates (≈68% higher than the control), and shortened lag phases for both acids, indicating accelerated and methanogenesis-dominated AD performance.

Acetic acid markedly increased SB-EPS polysaccharides up to 34.10 ± 0.34 mg eq Glc mL^−1^ and promoted protein redistribution toward SB- and LB-EPS at higher dosages, indicating effective disruption of the rigid EPS matrix. In contrast, citric acid mainly affected outer EPS layers, increasing LB-EPS biomolecules at low-to-moderate dosages but showing weaker protein decomposition overall, consistent with its lower availability of undissociated protons and strong buffering and chelation capacity.

A total of 31 pharmaceuticals were detected in anaerobically digested sludge at concentrations ranging from <10 ng g^−1^ for most compounds to µg g^−1^ levels for ibuprofen (4–11 µg g^−1^) and up to 13,000 ng g^−1^ for ciprofloxacin in specific samples, reflecting differences in consumption patterns, hydrophobicity, and biodegradability. Acidic pretreatments did not significantly alter the overall concentration ranges of pharmaceuticals compared to the control, with only amisulpride and citalopram showing significantly lower concentrations after acetic acid pretreatment. Nevertheless, 12 compounds exhibited markedly lower concentrations after anaerobic digestion than in the substrate prior to AD, indicating that enhanced digestion primarily improved bulk organic matter conversion rather than the systematic removal of pharmaceutical micropollutants.

## Figures and Tables

**Table 1:** Maximum specific methane potential (M_∞_), maximum specific methane production rate (μmax), lag phase (λ) and coefficient of determination (R^2^) with standard deviation (triplicate) for the Gompertz kinetic model.

Parameter	λ (d)	µ_max_ (mL CH_4_ g^−1^ VS d^−1^)	M_∞_ (mL CH_4_ g^−1^ VS)	R^2^
Control	1.3 ± 0.1	5.1 ± 0.6	29.1 ± 0.5	0.999 ± 0.002
Acetic acid (5 mM)	0.8 ± 0.1	16.4 ± 0.2	104.1 ± 0.8	0.996 ± 0.001
Citric acid (0.1 g/gTS)	1.0 ± 0.1	16.2 ± 0.2	73.3 ± 2.8	0.998 ± 0.002

**Table 2:** Physicochemical properties of the substrate and inoculums used in this study.

Parameter	Substrate	Inoculum
pH	6.35 ± 0.09	7.12 ± 0.06
TS (%)	4.35 ± 0.05	0.53 ± 0.10
VS (%)	74.3 ± 0.12	86.7 ± 0.09
COD (mg/kg)	520,930 ± 274	601,254 ± 141
N (%)	3.36 ± 0.02	n.a.
C (%)	27.5 ± 0.04	n.a.
C/N ratio	8.18 ± 0.02	n.a.

n.a. not analyzed.

## Data Availability

Data will be made available on request.
